# Correlates of time spent walking and cycling to and from work: baseline results from the commuting and health in Cambridge study

**DOI:** 10.1186/1479-5868-8-124

**Published:** 2011-11-10

**Authors:** Jenna Panter, Simon Griffin, Andrew Jones, Roger Mackett, David Ogilvie

**Affiliations:** 1 Medical Research Council Epidemiology Unit, Institute of Metabolic Sciences, Cambridge, UK; 2 UKCRC Centre for Diet and Activity Research (CEDAR), Institute of Public Health, Cambridge, UK; 3 School of Environmental Sciences, University of East Anglia, Norwich, UK; 4 Centre for Transport Studies, University College London, London, UK

**Keywords:** transport, active commuting, environmental perceptions, distance, Theory of Planned Behaviour

## Abstract

**Purpose:**

Environmental perceptions and psychological measures appear to be associated with walking and cycling behaviour; however, their influence is still unclear. We assessed these associations using baseline data from a quasi-experimental cohort study of the effects of major transport infrastructural developments in Cambridge, UK.

**Methods:**

Postal surveys were sent to adults who travel to work in Cambridge (n = 1582). Questions asked about travel modes and time spent travelling to and from work in the last week, perceptions of the route, psychological measures regarding car use and socio-demographic characteristics. Participants were classified into one of two categories according to time spent walking for commuting ('no walking' or 'some walking') and one of three categories for cycling ('no cycling', '1-149 min/wk' and ' ≥ 150 min/wk').

**Results:**

Of the 1164 respondents (68% female, mean (SD) age: 42.3 (11.4) years) 30% reported any walking and 53% reported any cycling to or from work. In multiple regression models, short distance to work and not having access to a car showed strong positive associations with both walking and cycling. Furthermore, those who reported that it was pleasant to walk were more likely to walk to or from work (OR = 4.18, 95% CI 3.02 to 5.78) and those who reported that it was convenient to cycle on the route between home and work were more likely to do so (1-149 min/wk: OR = 4.60, 95% CI 2.88 to 7.34; ≥ 150 min/wk: OR = 3.14, 95% CI 2.11 to 4.66). Positive attitudes in favour of car use were positively associated with time spent walking to or from work but negatively associated with cycling to or from work. Strong perceived behavioural control for car use was negatively associated with walking.

**Conclusions:**

In this relatively affluent sample of commuters, a range of individual and household characteristics, perceptions of the route environment and psychological measures relating to car use were associated with walking or cycling to and from work. Taken together, these findings suggest that social and physical contexts of travel decision-making should be considered and that a range of influences may require to be addressed to bring about behaviour change.

## Introduction

Promoting physical activity in adults is a public health priority [1]. Moderate intensity activity has significant cardiovascular [2] and mental health [3] benefits, and protects against osteoporosis [4], obesity, and related disorders [5]. Some evidence suggests that particular types of moderate intensity activities, such as walking and cycling, may be associated with positive health outcomes. For example, adults who regularly walk or cycle to work have higher levels of cardio-respiratory fitness than those who do not [6] and commuter cyclists have a lower mortality risk than non-cycling commuters, independent of leisure time physical activity [7]. Furthermore, promoting walking and cycling is likely to be associated with wider societal benefits such as reductions in traffic volume and congestion, air pollution, and carbon emissions [8].

Ecological models of health related behaviour [9] and evidence from the existing literature [10,11] suggest that factors pertaining to both individuals and their environments may be important correlates of walking and cycling for transport ('active travel') and walking and cycling to get to and from work in particular ('active commuting'). Amongst the few studies which have assessed the relative importance of factors at both levels, the findings are inconsistent. Some studies report that both psychosocial or psychological and environmental explanatory variables are associated with walking and cycling for transport [12-14]. For example, de Bourdeaudhuij and colleagues [12] found that higher land use mix and social support were associated with active travel. Others, such as Lemieux and Godin [14], report that only psychological factors, namely positive intentions and strong habits for walking and cycling, were significant. However, few studies have considered the correlates of walking and cycling separately; this may be important because the characteristics of an environment which encourage walking may be quite different to those which support cycling [15]. Furthermore, most studies have only examined environmental factors related to the residential neighbourhood; this may be important because it neglects the potential influence of the characteristics of the entire route between home and work [16]. Route based perceptions - by their very nature - capture components of the origin, route and destination and only a few studies have explored the associations between adults' perceptions of the journey to work and their commuting behaviour [13]. If exposures and behaviours are not sufficiently specific [17], the results of analyses may be misleading, hindering a clear understanding of the influences on both behaviours. We also hypothesised that the associations between putative explanatory variables and walking and cycling may differ according to car access, as car access imposes significant constraints on the choice of travel mode. In this study we report the associations between psychological factors, perceptions of the environment on the route, and walking and cycling to and from work in a survey of adults who travel to work in Cambridge, UK.

## Methods

### Study design and sample

We examined cross-sectional data from participants taking part in the first phase of the *Commuting and Health in Cambridge* study in Cambridge, UK. This is a quasi-experimental study of the effects of a major transport infrastructural intervention on travel behaviour, physical activity and related wider health impacts. The methods of recruitment and sampling for the study have been described in detail in a full study protocol paper [18]. In summary, adults over the age of 16 working in Cambridge and living within a radius of approximately 30 km of Cambridge city centre were invited to participate through a predominantly workplace-based recruitment strategy. Many of the workplaces were members of the Cambridgeshire Travel for Work partnership and all were located within Cambridge, however there was heterogeneity in their geographical setting, which spanned city centre and urban fringe locations. A range of types of workplaces were invited, including local authorities, healthcare providers, retail outlets as well as higher and further education institutions. Potential participants registered their interest with the study team; those deemed eligible received a postal questionnaire and returned it in the freepost envelope provided. The questionnaire asked about travel to and from work in the last seven days, psychological measures related to car use and perceptions of the environment on the route between home and work, as well as a range of other individual and socio-demographic factors (Additional file 1). Data included in these analyses were collected between May and November 2009. Ethical approval for the study was obtained from the Hertfordshire Research Ethics Committee and written informed consent was provided by each participant.

### Travel to and from work

In the absence of a valid measure of travel behaviour, the use of all travel modes on the journey to and from work on the last seven days was assessed using a one-page instrument adapted from one used previously and shown to have acceptable test-retest reliability [19]. Two questions were included to assess (i) whether participants ever travelled by bicycle part or all of the way to work and (ii) the typical duration of the cycling stage of the journey (in minutes). Two analogous questions were asked for walking. From these responses, the total times spent travelling to and from work by bicycle and on foot in the last seven days were calculated. Data on the duration of time spent walking and/or cycling were only used in this estimation if participants had reported walking or cycling in the last seven days. As the distribution of time spent walking in the sample was highly skewed with a large number of participants reporting no walking in the last seven days, participants were classified as either engaging in either 'no walking' or 'some walking' on the journey to and from work. Similarly, a number of participants also reported spending no time cycling to and from work, and based on the distribution of the data three categories were created: 'No cycling in the last week', '1-149 minutes per week' and '150 minutes per week or more'. The upper cutpoint approximately equates to 30 minutes of cycling on five days of the week, reflecting current guidelines for recommended levels of physical activity in adults [20].

### Perceptions of the route between home and work

As travel to and from work was the primary behaviour of interest in this study, perceptions of the characteristics of the route between home and work were assessed. The few previous studies on this topic have suggested that self-reported perceptions of routes appear valid when compared with reports from an expert panel [21] and that route-based perceptions are associated with cycling behaviour [22]. In this study, participants reported their level of agreement with seven statements that could be used to describe the environment along their route to and from work using a five-point Likert scale. These statements were selected from a longer list of items used previously and shown to have acceptable test-retest reliability [23,24] but were applied in this study to participants' routes to work rather than to their residential neighbourhoods. Responses to these items were collapsed such that those who 'strongly agreed' or 'agreed' with an item were compared with those who 'strongly disagreed', 'disagreed' or 'neither disagreed or agreed'. The distribution of responses is shown in the additional file (Additional file 2). Sensitivity analysis showed that grouping 'neither disagreed or agreed' with 'strongly agreed' and 'agreed' made no significant difference to the results.

Participants also reported the distance between home and work in either kilometres or miles. Although the accuracy of self-reported distances is generally poor, the distances of commuting trips tend to be more accurately reported than those of other types of trip because they are made regularly [25]. We were confident that participants would be able to identify very long or short trips, and therefore derived two binary variables for distance. The first classified participants as living less or more than 3 km from work (representing a notional maximum acceptable walking distance), and the second as living less or more than 5 km from work (representing a notional maximum acceptable cycling distance) [26].

### Psychological measures related to car use

A previously validated questionnaire [27] which measures perceived behavioural control (PBC), intention, instrumental attitude, affective attitude and subjective norms was applied to car use, using two items per construct. Car use was chosen as the behavioural reference for these questions to reflect the wider interest of the study in exploring the potential for promoting a modal shift from car travel to more sustainable modes of transport [18]. Respondents reported their agreement with each statement using a five-point Likert scale, from which mean scores within each construct (pair of items) were calculated and classified into tertiles.

Habit strength for using the car to travel to and from work was assessed using participants' reported agreement with seven statements on a five-point Likert scale. These statements were derived from the Habit Strength Index [28], which assesses self-identity and automaticity of behaviour, has been shown to have high test-retest and internal reliability [28,29] and has been validated against other measures of habit strength [30]. We found that the Cronbach's alpha (internal consistency) of the seven items used here was 0.97, but for the purposes of the current cross-sectional analysis we chose to exclude two items representing frequency and history of past behaviour, as these may have artificially strengthened the apparent associations between habit and walking or cycling to work [31]. We therefore derived a summary habit score in favour of car use based on the five remaining items (automaticity, requiring effort not to do, belonging to a daily routine, finding it hard not to do, being typically me), for which Cronbach's alpha (0.95) was very similar to that for the seven items. As the distribution of mean habit scores for car use (range 1-5) was skewed with 40% of participants reporting a mean score of 1, a binary summary variable was created to distinguish those who mean score was equal to 1 from those whose mean score was greater than 1.

### Individual and household characteristics

Characteristics of participants and their households were assessed including date of birth, gender, educational qualifications, housing tenure, household composition, access to cars and bicycles, possession of a driving licence, presence of long-term limiting illness or disability, difficulty walking and self-reported height and weight. Body mass index (BMI) was calculated by dividing weight by height squared (kg/m^2^) and for the purposes of analysis, participants were assigned to one of three categories of weight status; 'normal or underweight', 'overweight' or 'obese' based on internationally recognised cut offs [32].

Objective measures of urban-rural status were estimated within a Geographical Information System (GIS) whereby each participant's home postcode was converted into a map location using Code-Point, a dataset that identifies the centre point for all postcodes in Great Britain [33]. Urban-rural status of the home location was defined using the Urban and Rural Classification [34] of the Output Area (OA) within which the participant was resident and the available categories were collapsed into 'urban' and 'rural', as there were only small numbers of participants in several of the more rural groupings.

### Statistical analyses

Descriptive data were summarized using percentages. The explanatory variables used in these analyses were chosen for study because they were considered to be conceptually important and had some theoretical support in the literature [9,35]. Univariate associations between putative explanatory variables (individual and household characteristics, route perceptions and psychological measures towards car use) and walking and cycling behaviour were assessed using logistic and multinomial logistic regression models respectively to estimate the odds of walking to work and, separately, the odds of spending 1-149 minutes per week or 150 minutes or more per week cycling to work. For walking, the following self-reported environmental characteristics were tested: pleasantness of route, convenience for walking, convenience of public transport, safety for crossing the road, traffic volume and the 3 km distance variable. For cycling, convenient routes for cycling, safety for crossing the road, traffic volumes, traffic danger for cyclists and the 5 km distance variable were tested. Those putative explanatory variables for which a significance of p < 0.25 [36] was obtained in univariate analysis were carried forward for entry into multiple regression models, initially restricted to the individual and household explanatory variables. Non-significant variables (p > 0.05) were then individually removed from these models and the models re-fitted each time. Interaction terms were also created to represent interactions between car ownership and each of the other individual and household explanatory variables. These were tested by adding the terms one at a time, after which the model was re-fitted including only the significant variables (Model A).

We then examined the influence of the combined individual, household and psychological factors in one model (Model B1) and the influence of the combined individual and household factors, route perceptions and urban-rural status in a second model (Model B2) using the model building techniques described above. We chose this strategy for a combination of reasons. First, having identified some collinearity between the presence of limiting long term illness or disability and the psychological variables reflecting attitudes and perceived behavioural control, we nonetheless considered it important to model these explanatory variables simultaneously because of their importance in influencing behaviour in the general population. Second, because stepwise regression modelling may exacerbate collinearity between variables [37] we considered it more appropriate to treat the psychological measures related to car use and the perceptions of the route environment in two separate models. As a guide to the interpretation of the model outputs, we have presented pseudo-R^2 ^values to enable comparisons with previous literature in this area. However, these values should not be interpreted as being equivalent to R^2 ^values from ordinary least squares regression models. All analyses were conducted in Stata 11.1.

## Results

### Sample characteristics

Of the 1582 participants who were sent a questionnaire, 1164 (74%) provided consent and returned a completed questionnaire. Of these, 22 were excluded as they reported no journeys to or from work in the last seven days, leaving 1142 completed responses for analysis. Compared to those included in the analysis, non-responders were more likely have at least one car in the household (98.2% versus 85.2%, p = 0.001) but did not differ significantly from responders in terms of age, gender or urban-rural status.

There were few missing responses to most of the questions, with the exception of those asking the number of children under the age of 5 and the number between 5 and 15 years. We combined the responses to both of these items into one binary variable representing the presence of at least one child in the household, on which 785 participants could be clearly categorised on the basis of their responses. We replaced missing responses with zero in the remaining participants (n = 357) and we repeated the analysis using only those who positively reported zero children in the household. This yielded no substantive alterations in the effect size or statistical significance of the explanatory variables. Table 1 gives further details of the characteristics of the sample according to whether or not they reported any walking or any cycling to or from work in the last seven days.

**Table 1:** Individual and household characteristics of the sample according to time spent walking and cycling to work

	Percentage (number)					
		Time spent walking* (n = 1128)		Time spent cycling* (n = 1133)		
	All participants (n = 1143) % (number)	None (n = 801)	Some (n = 327)	None (n = 538)	1-149 mins (n = 276)	> 150 mins (n = 319)
**Individual characteristics**						
Gender (n = 1143)						
Male	31.5 (360)	78.7 (286)	21.3 (77)	35.1(125)	29.2 (104)	35.7 (127)
Female	68.5 (783)	66.6 (536)	33.3 (250)	53.1 (413)	22.2 (172)	24.7 (192)
Age (n = 1143)						
< 30	16.4 (188)	65.6 (122)	34.4 (64)	42.0 (78)	27.4 (51)	30.6 (57)
30-40	28.6 (327)	71.8 (231)	28.2 (91)	45.2 (147)	25.3 (82)	29.5 (96)
40-50	25.9 (297)	74.0 (216)	26.0 (76)	47.8 (141)	23.4 (69)	28.8 (85)
50-60	21.3 (244)	72.3 (176)	27.7 (67)	51.0 (124)	22.4 (54)	26.6 (64)
Over 60 years	7.6 (87)	65.8 (56)	34.1 (29)	56.5 (48)	23.5 (20)	20.0 (17)
Highest educational qualification (n = 1136)						
Less than degree	28.1 (319)	69.5 (219)	30.5 (96)	61.8 (196)	18.4 (58)	19.8 (63)
Degree or higher	71.9 (817)	71.8 (579)	28.2 (227)	41.7 (338)	26.7 (216)	31.6 (255)
Weight status (n = 1125)						
Normal or underweight	62.8 (707)	71.8 (503)	28.2 (197)	41.3 (289)	26.7 (186)	32.0 (223)
Overweight	27.6 (310)	68.3 (209)	31.7 (97)	52.3 (162)	23.5 (73)	24.2 (75)
Obese	9.6 (108)	72.1 (75)	27.9 (29)	72.0 (77)	12.2 (13)	15.8 (17)
Difficulty walking (n = 1141)						
No	98.6 (1123)	70.9 (786)	29.1 (322)	47.2 (546)	24.3 (271)	28.5 (317)
Yes	1.5 (18)	76.5 (14)	23.5 (4)	62.5 (11)	31.2 (5)	6.3 (1)
Long term illness (n = 1159)						
No	89.9 (1024)	71.9 (727)	28.1 (284)	45.7 (465)	24.7 (251)	29.6 (301)
Yes	10.1 (115)	62.5 (71)	37.5 (42)	70.0 (72)	22.5 (25)	13.5 (15)
Driving licence (n = 1142)						
No Yes	9.6 (110) 90.4 (1032)	46.4 (51) 73.6 (749)	53.6 (59) 26. 4(268)	55.5 (61) 46.6 (477)	21.8 (24) 24.7 (252)	22.7 (25) 28.7 (293)

**Household characteristics**						
Number of children in the						
household (n = 1142)						
None	80.0 (913)	69.4 (627)	30.6 (276)	48.8 (442)	23.0 (208)	28.2 (255)
One or more	20.0 (229)	77.2 (173)	22.8 (51)	41.9 (95)	29.9 (68)	28.2 (64)
Home ownership (n = 1139)						
Does not own home	27.2 (310)	65.8 (202)	34.2 (105)	40.9 (126)	27.9 (86)	31.2 (96)
Home owner	72.8 (829)	72.9 (596)	27.1 (221)	49.8 (409)	23.1 (189)	27.1 (223)
Number of cars in household (n = 1143)						
None	14.7 (169)	58.3 (98)	41.7 (70)	36.4 (60)	29.7 (49)	33.9 (56)
One car or more cars	85.3 (974)	73.2 (364)	26.8 (142)	49.3 (477)	23.5 (227)	27.2 (263)
Distance to work from home (n = 1142)						
0-3.0 km	12.4 (142)	52.5 (74)	47.5 (67)	35.2 (50)	50.7 (72)	14.1 (20)
3.1-5.0 km	26.5 (302)	79.2 (236)	20.8 (62)	17.5 (52)	40.3(120)	42.2 (126)
5.1-10.0 km	19.0 (216)	78.0 (167)	22.0 (47)	32.1 (68)	12.8 (27)	55.1 (117)
Over 10 km	42.1 (482)	69.8 (324)	30.2 (150)	74.0 (368)	11.9 (56)	14.1 (56)
Home location (n = 1142)						
Urban	65.8 (752)	68.2 (514)	30.8 (229)	37.7 (281)	29.9 (222)	32.4(242)
Non-Urban	34.2 (390)	74.4 (286)	25.6 (98)	66.3 (257)	14.0(54)	19.7 (76)

*Row percentages and number of participants within each category are given

### Explanatory variables associated with walking

In terms of individual and household characteristics, women were almost twice as likely to walk and those without access to a car and who lived less than 3 km from work were three times more likely to walk (odds ratio (OR) 3.39, 95% CI 1.86 to 6.17), whereas those who had a driving licence were less likely to walk to or from work (OR 0.38 95%CI 0.25 to 0.60; Table 2, Model A). The pseudo-R^2 ^value indicated that 4% of variance in walking behaviour was explained by these factors alone. Adjusting for individual and household variables, those with higher perceived behavioural control over car use were half as likely to walk (OR 0.53, 95% CI 0.34 to 0.83), whereas stronger attitudes in favour of car use were associated with an increased likelihood of walking (OR 1.46 95%CI 0.98 to 2.16; Table 2, Model B1). The inclusion of these factors resulted in a small increase in the variance explained by the model, bringing the total to 5%. Participants who reported that it was pleasant to walk (OR 4.18, 95% CI 3.02 to 5.78) and that there was convenient public transport were more likely to report walking, yet those who reported little traffic were less likely to report walking (OR 0.38, 95% CI 0.21 to 0.68) (Table 2; Model B1 and B2). As a result of the inclusion of these variables, the variance in walking behaviour explained by the model increased to 13%. In order to aid interpretation of the odds ratios for the interaction terms, the analysis was then stratified by car access ('no access to a car' and 'access to a car'; Table 3). Distance showed stronger negative associations with walking in those without access to a car than those with a car, whereas the associations observed between walking and psychological measures of car use (Model B1) and perceptions of the route environment (Model B2) were significant in the group with access to a car.

**Table 2:** Multiple regression models for the odds of spending any time walking to work

	Model A OR (95% CI) p	Model B1 OR (95% CI) p	Model B2 OR (95% CI) p
Individual and household characteristics			
Gender (reference: male)			
Female	1.85 (1.36-2.51) 0.001	1.81 (1.33-2.47) 0.001	1.82 (1.32-2.51) 0.001
Driving licence (reference: no)			
Yes	0.38 (0.25-0.60) 0.001	0.41 (0.26- 0.65) 0.001	0.47 (0.29-0.76) 0.001
Car access × Distance (reference: access to a car, lives ≥ 3 km from work)			
Owns a car, lives < 3 km from work	1.99 (1.25-3.19) 0.003	2.00 (1.22-3.29) 0.006	1.54 (0.93-2.56) 0.113
No car, lives ≥ 3 km from work	1.07 (0.67-1.70) 0.757	0.98 (0.60-1.59) 0.942	0.86 (0.52-1.38) 0.740
No car, lives < 3 km from work	3.39 (1.86-6.17) 0.001	3.21 (1.71-6.02) 0.001	2.31 (1.24-4.29) 0.001

Psychological measures of car use			
Intention score (reference: low intention)			
Mid intention score	-	n.i	-
High intention score			
Attitude score (reference: low attitude)			
Mid attitude score	_	1.07 (0.72-1.58) 0.712	_
High attitude score		1.46 (0.98-2.16) 0.050	
PBC score (reference: low PBC)			
Mid PBC score	-	0.82 (0.57-1.20) 0.322	-
High PBC score		0.53 (0.34-0.83) 0.006	
Social norm score (reference: low social norm)			
Mid social norm score	-	n.i	-
High social norm score			
Habit score (reference: low habits)			
High habit score	-	n.i	-

Perceptions of the route environment ^a^			
It is pleasant to walk	-	-	4.18 (3.02-5.78) 0.001
There is convenient public transport	-	-	1.46 (1.10-1.96) 0.010
There is little traffic	-	-	0.38 (0.21-0.68) 0.001
There are no convenient routes for walking	-	-	n.i
It is safe to cross the road	-	-	n.i
Urban-rural Status (reference: urban)			
Rural	-	-	n.i

CI confidence intervals; p p-value; PBC Perceived behavioural control; - not entered into the model, n.i not included; ^a ^the reference category is strongly disagree, disagree or neither disagree nor agree.

**Table 3:** Multiple regression models for odds of engaging in any walking to work stratified according to car availability within the household

	Model B1		Model B2	
	No car OR (95% CI) p	Car OR (95% CI) p	No car OR (95% CI) p	Car OR (95% CI) p
Individual and household characteristics				
Gender (reference: male)				
Female	2.18 (1.03-4.64) 0.041	1.74 (1.24-2.44) 0.001	1.96 (0.92-4.17) 0.080	1.80 (1.26-2.56) 0.005
Driving licence (reference: no) Yes	0.37 (0.18-0.74) 0.005	0.44 (0.23-0.81) 0.010	0.40 (0.19-0.81) 0.012	0.54 (0.28-1.02) 0.060
Distance (reference: lives < 3 km from work)				
Lives ≥ 3 km from work	0.28 (0.13-0.60) 0.001	0.48 (0.29-0.79) 0.004	0.36 (0.17-0.77) 0.009	0.63 (0.38-1.06) 0.08

Psychological measures of car use				
Attitude score (reference: low attitude)				
Mid attitude score	0.60 (0.23-1.59) 0.313	1.19 (0.77-1.84) 0.409	-	-
High attitude score	1.73 (0.61-4.88) 0.300	1.50 (0.97-2.32) 0.067		
PBC score (reference: low PBC)				
Mid PBC score	0.50 (0.19-1.30) 0.158	0.89 (0.59-1.35) 0.599	-	-
High PBC score	0.55 (0.03-8.29) 0.669	0.56 (0.35-0.90) 0.017		

Perceptions of the route environment ^a^				
It is pleasant to walk	-	-	4.73 (1.67-13.37) 0.003	4.12 (2.93-5.81) 0.001
There is convenient public transport	-	-	1.66 (0.82-23.36) 0.158	1.45 (1.05-1.99) 0.022
There is little traffic	-	-	0.44 (0.15-1.50) 0.211	0.36 (0.18-0.72) 0.004

CI confidence intervals; p p-value; PBC Perceived behavioural control; - not entered into the model; ^a ^the reference category is strongly disagree, disagree or neither disagree nor agree.

### Explanatory variables associated with cycling

Table 4 shows the odds of cycling 1-149 minutes and 150 minutes or more over the last seven days using the same model structures (Models A-B2). Women (OR 0.52, 95% CI 0.33 to 0.82) and obese individuals (OR 0.31, 95% CI 0.14 to 0.70) were less likely to cycle 1-149 minutes per week, whereas those with a degree level education (OR 1.28, 95% CI 0.80 to 2.04) and those with at least one child in the household (OR 2.11 95% CI 1.34 to 3.32) were more likely to report cycling 1-149 minutes per week (Model A). The pseudo-R^2 ^value indicated that 12% of variance in cycling behaviour was explained by these factors alone. The individual and household characteristics associated with cycling 1-149 minutes and 150 minutes or more were similar with the exception of limiting long term illness or disability, whereby those with a limiting long term illness were half as likely to cycle 150 minutes or more per week, compared to those without (OR 0.42, 95% CI 0.19 to 0.88), but no association was evident for those cycling 1-149 minutes per week. Those with stronger attitudes in favour of car use were less likely to cycle (OR 0.28, 95% CI 0.16 to 0.49) and those who reported convenient routes for cycling were more likely to cycle (OR 4.60, 95% CI 2.88 to 7.34) (Models B1 and B2). The addition of psychological and environmental factors to the model resulted in 17% of the variance in cycling behaviour being explained in both models. Habits for car use were not shown to be important explanatory variables for cycling behaviour and urban-rural status was not associated with walking or cycling, after adjustment for individual or household characteristics, hence these results are not presented. As with the results for observed for walking, many of the significant overall associations for personal (in particular: gender, weight status, and distance to work), psychological (attitudes) and environmental (perceived convenience) explanatory variables observed in the sample for cycling behaviour were largely accounted for by the group who had access to a car (Table 5).

**Table 4:** Multiple regression models for odds of spending ≤ 149 minutes and ≥ 150 minutes of cycling to and from work

	Model A		Model B1		Model B2	
	1-149 mins cycling OR(95% CI) p	> 150 mins cycling OR(95% CI) p	1-149 mins cycling OR(95% CI) p	> 150 mins cycling OR(95% CI) p	1-149 mins cycling OR(95% CI) p	> 150 mins cycling OR(95% CI) p
Individual and household characteristics						
Gender (reference: male)						
Female	0.52 (0.33-0.82) 0.009	0.44 (0.29-0.66) 0.001	0.52 (0.32-0.82) 0.006	0.44 (0.28-0.67) 0.001	0.51 (0.32-0.81) 0.005	0.44 (0.29-0.66) 0.001
Highest educational qualification (reference: less than degree)						
Degree or higher	1.28 (0.80-2.04) 0.057	1.77 (1.15-2.72) 0.009	1.22 (0.75-1.97) 0.414	1.74 (1.10-2.75) 0.016	1.35 (0.83-2.20) 0.221	1.80 (1.15-2.80) 0.009
Weight status (reference: normal weight)						
Overweight	0.55 (0.34-0.90) 0.019	0.57 (0.37-0.89) 0.014	0.57 (0.34-0.94) 0.028	0.59 (0.37-0.93) 0.026	0.54 (0.32-0.89) 0.018	0.57 (0.36-0.90) 0.018
Obese	0.31 (0.14-0.70) 0.005	0.38 (0.19-0.76) 0.011	0.36 (0.16-0.81) 0.014	0.48 (0.23-1.00) 0.052	0.32 (0.14-0.73) 0.007	0.39 (0.19-0.81) 0.019
Limiting long term illness (reference: no) Yes	1.11 (0.57-2.16) 0.752	0.42 (0.19-0.88) 0.022	1.27 (0.64-2.55) 0.485	0.53 (0.24-1.17) 0.122	1.11 (0.55-2.25) 0.756	0.44 (0.20-0.94) 0.036
Number of children (reference: none) One or more	2.11 (1.34-3.32) 0.001	1.40 (0.93-2.14) 0.112	2.00 (1.25-3.18) 0.003	1.29 (0.83-2.02) 0.253	2.31 (1.45-3.70) 0.001	1.46 (0.95-2.26) 0.084
Car access × Distance (reference: access to a car lives ≥ 5 km from work)						
Car, lives < 5 km from work	13.9 (8.33-23.19) 0.001	5.05 (3.07-8.30) 0.001	9.24 (5.38-15.88) 0.001	3.15 (1.85-5.38) 0.001	10.29 (6.07-17.4) 0.001	4.00 (2.41-6.65) 0.001
No car, lives ≥ 5 km from work	1.19 (0.32-4.42) 0.789	3.28 (1.44-7.47) 0.005	0.73 (0.19-2.82) 0.655	1.99 (0.83-4.79) 0.122	1.06 (0.28-4.08) 0.922	2.93 (1.25-6.88) 0.013
No car, lives < 5 km from work	4.41 (2.36-8.51) 0.001	2.50 (1.41-4.42) 0.002	2.34(1.18-4.64) 0.015	1.27 (0.67-2.41) 0.449	3.31 (2.88-6.31) 0.001	2.01 (1.12-3.62) 0.019

Psychological measures of car use						
Intention (ref: low intention)						
Mid intention score	-	-	n.i	n.i	-	-
High intention score						
Attitude (ref: low attitude)						
Mid attitude score	-	-	0.73 (0.40-1.31) 0.301	1.29 (0.76-2.17) 0.335	-	-
High attitude score			0.28 (0.16-0.49) 0.001	0.19 (0.11-0.33) 0.001		
PBC (ref: low PBC)						
Mid PBC score	-	-	n.i	n.i	-	-
High PBC score						
Social norm (ref: low social norm) Mid social norm score High social norm score	-	-	n.i	n.i	-	-
Habit score (reference: low habits)						
High habit score	-	-	n.i	n.i		

Perceptions of the route environment ^a^						
The roads are dangerous for cyclists	-	-	-	-	n.i	n.i
There are convenient routes for cycling	-	-	-	-	4.60(2.88-7.34) 0.001	3.14 (2.11-4.66) 0.001
There is little traffic	-	-	-	-	n.i	n.i
It is safe to cross the road	-	-	-	-	n.i	n.i
Urban-rural Status (reference = urban)						
Rural	-	-	-	-	n.i	n.i

CI confidence intervals; p, p value; PBC Perceived behavioural control; - not entered into the model; n.i, not included in the model; ^a ^the reference category is strongly disagree, disagree or neither disagree nor agree

**Table 5:** Multiple regression models for odds of engaging in < 149 minutes and ≥ 150 minutes of cycling to and from work, stratified according to car availability within the household

	Model B1: Personal & psychological factors				Model B2: Personal & environmental factors			
	No car		Car		No car		Car	
	1-149 mins cycling OR(95% CI) p	> 150 mins cycling OR(95% CI) p	1-149 mins cycling OR(95% CI) p	> 150 mins cycling OR(95% CI) p	1- 149 mins cycling OR(95% CI) p	> 150 mins cycling OR(95% CI) p	1- 149 mins cycling OR(95% CI) p	> 150 mins cycling OR(95% CI) p
**Personal factors**								
Gender (ref = male)								
Female	0.67 (0.22-2.01) 0.486	0.58 (0.22-1.48)0.258	0.46 (0.27-0.78)0.004	0.41 (0.25-0.66)0.001	0.72 (0.24-2.14)0.566	0.54 (0.22-1.36)0.196	0.45 (0.26-0.76)0.003	0.41 (0.26-0.66) 0.001
Highest educational qualification (ref = < degree)								
Degree or higher	1.25 (0.32-4.87) 0.741	1.46 (0.45-4.67)0.459	1.19 (0.71-2.00)0.500	1.70 (1.07-2.94)0.035	1.24 (0.31-4.81)0.755	1.18 (0.38-3.61)0.767	1.36 (0.80-2.32)0.247	1.94 (1.18-3.17) 0.008
Weight status (ref = normal weight)								
Overweight	0.41 (0.09-1.85)0.248	0.68 (0.20-2.27)0.538	0.55 (0.32-0.95)0.034	0.54 (0.32-0.90)0.020	0.41 (0.09-1.83) 0.244	0.60 (0.18-1.94) 0.402	0.53 (0.30-0.92)0.024	0.54 (0.33-0.89) 0.017
Obese	0.59 (0.04-7.28)0.681	2.23 (0.35-13.84)0.395	0.34 (0.14-0.83)0.018	0.38 (0.16-0.89)0.026	0.65 (0.05-7.86) 0.735	1.73 (0.28-10.4) 0.545	0.29 (0.11-0.71)0.007	0.30 (0.13-0.69) 0.005
Limiting illness (ref = no) Yes	0.71 (0.14-3.59)0.681	0.48 (0.10-2.20)0.352	1.48 (0.68-3.23)0.314	0.51 (0.19-1.34)0.174	0.78 (0.15-4.10) 0.774	0.54 (0.12-2.34) 0.416	1.22 (0.55-2.69)0.620	0.38 (0.15-0.97) 0.045
School aged children (ref = none) More than one	16.07(0.14-166.7) 0.02	7.20 (0.67-77.18)0.103	1.75 (1.07-2.85)0.023	1.21 (0.76-1.94)0.413	14.63 (1.47-145.7) 0.02	5.55 (0.12-56.4)0.147	2.06 (1.26-3.39)0.004	1.40 (0.89-2.21) 0.143
Car ownership (ref = no) yes	-	-	-	-	-	-	-	-
Distance (ref = < 5 km) > 5 km	0.31 (0.07-1.32)0.114	1.39 (0.51-3.81)0.511	0.11 (0.06-0.19)0.001	0.33 (0.19-0.58)0.001	0.31 (0.07-1.31)0.114	1.33 (0.50-3.49) 0.558	0.09 (0.05-0.16)0.001	0.25 (0.15-0.42) 0.001
**Psychological measures of car use**								
Attitude (ref = low attitude)								
Mid attitude score	1.27 (0.28-5.73)0.754	4.58 (1.38-15.21) 0.013	0.61 (0.31-1.19)0.151	0.91 (0.49-1.66)0.763				
High attitude score	0.48 (0.10-2.18)0.348	0.56 (0.16-1.96)0.372	0.24 (0.12-0.44)0.001	0.14 (0.08-0.26)0.001				
**Perceptions of the route environment ^a^**								
There are convenient routes for cycling					2.14 (0.71-6.45) 0.173	1.91 (0.77-4.76)0.160	5.37 (3.20-9.02)0.001	3.51 (2.26-5.47) 0.001

CI, Confidence Intervals; p, p value; n.i, not included in the model; ^a ^the reference category is strongly disagree, disagree or neither disagree nor agree

## Discussion

### Principal findings

In this study, a range of individual and household characteristics were associated with an increased likelihood of spending time walking or cycling to and from work, in particular having a relatively short distance between home and work and not having access to a car. Together this group of explanatory variables was more strongly associated with cycling behaviour than perceptions of the route between home and work or psychological measures related to car use, of which only a few variables remained significant in multiple regression models. In contrast, individual and household characteristics predicted a relatively small amount of variance in walking to and from work, whereas perceptions of the route environment accounted for a larger proportion of the variance in this behaviour. Many of the associations between explanatory variables and walking and cycling behaviour were stronger in the group with access to a car.

### Strengths and limitations

Our study has a number of strengths and limitations. Strengths include the separate analyses of the explanatory variables associated with walking and cycling, the large sample size of predominantly healthy working adults commuting from both urban and rural areas, and the use of reliable measures to assess perceptions of the environment and relevant psychological constructs, albeit applied in this study to a particular environmental context (the route to work) and a particular type of travel behaviour (car use) respectively. We also used time-based measures of travel to and from work which are more detailed than the more commonly used measures of 'usual' or 'main' mode. On the other hand, we cannot assume that walking or cycling were undertaken as sole modes of travel because the use of combinations of travel modes within one journey is relatively common in Cambridge [38]. In this sample, 28% of participants reported using a combination of travel modes on their journey to or from work at least once in the last seven days, for example by using public transport in combination with walking, or by driving part of the way and cycling the remainder.

Data were collected over a six month period between May to November to reduce the confounding effect of seasonal variations in travel behaviour, and as a result the reported levels of walking and cycling to work in this study may be higher than average in this setting. Participants reported the characteristics and conditions on or along their route to work; however, data were not available on the perceived supportiveness of the neighbourhood environment, which may also be important. Rather than asking participants to complete a battery of psychological measures relating to each of several potential modes of transport, we chose to focus on psychological measures regarding car use because the longitudinal study aims to assess whether changes to the environment designed to promote a shift away from car use are associated with changes in travel behaviour. In particular, we intend to assess the mechanisms underlying such changes and whether these operate via changes in, for example, attitudes towards car use. However, one consequence of this longitudinal focus is that these measures may not be optimally matched to all the possible behavioural summary measures considered in baseline analysis. Another limitation is that our sample contains a higher proportion of participants educated to degree level and a smaller proportion of obese adults than the general population of Cambridgeshire [39], no doubt reflecting the focus of this particular study on the predominantly healthy working adult population.

Cambridge is a city known for its cycling culture and its relatively high prevalence of cycling [39]. Nonetheless, comparison with local travel-to-work survey data suggests that cyclists may have been over-represented in our sample (47% versus 21% [38]). However, the local survey used a much cruder measure of travel behaviour and sampled participants from workplaces across the county, whereas our sample was drawn from workplaces in the city. Since higher levels of cycling tend to be observed in urban areas than rural areas and nearly half of our participants both lived and worked in Cambridge, these differences in sampling and data collection may account for the differences in the apparent prevalence of cycling, which would limit any concerns regarding potential selection bias. Whilst these relatively high levels of cycling observed allowed us to explore the explanatory variables associated with walking and cycling separately, the relative importance of the environmental and psychological factors may not be mirrored in other contexts where cycling is less popular or less embedded in social practices, and therefore the generalisability of these findings to these contexts may be limited. On the other hand, more than 85% of our participants also live in households with access to a car, and understanding the reasons why people choose, or do not choose, to walk or cycle despite having access to a car may be important for the development of strategies to promote active travel more generally.

### Relative contribution of explanatory variables

Individual and household characteristics accounted for a larger proportion of the variation in cycling behaviour than the other putative explanatory variables. This is generally similar to findings reported elsewhere [13,23,40], however we also found that environmental perceptions were associated with walking behaviour. In this study, many of these individual characteristics, such as educational level and BMI, were associated with cycling but not with walking. Positive associations between educational status and cycling behaviour have been reported previously [40] and given that ours was a relatively healthy and well educated sample, it is possible that these associations may be stronger in the population at large. One possible mechanism for the observed association is that owing to the high cost of housing in Cambridge city and its immediate surroundings, those who are well educated and have higher levels of disposable income may be more likely to be able to afford to live closer to work and therefore more likely to have the option of cycling. Due to the relatively small numbers of participants who reported walking and who had lower levels of education in this sample, the analysis may have been underpowered to detect comparable associations for walking.

Consistent with a systematic review of the environmental determinants of physical activity - which found that three quarters of all associations tested returned evidence consistent with the null hypothesis[41] - in this analysis, few of the environmental perceptions remained significant in final models. Of these, perceptions that it was pleasant to walk and that convenient public transport was available on the route to work were associated with walking, and the perceived convenience of cycle routes was associated with cycling. It is not possible to be sure from these results whether convenient routes facilitate cycling, or if cyclists are simply more aware than non-cyclists of the presence of convenient routes. In the context of this study, the provision of facilities which improve the convenience, quality and pleasantness of walking and cycling, such as traffic free routes, may be an important component of a broader intervention to promote active commuting, particularly if improvements are focused on routes which are frequently used, connect home and work locations within an acceptable cycling distance, and intersect with public transport stops.

We also found that participants who reported little traffic were less likely to spend time walking to work. This may reflect greater awareness and reporting of traffic in walkers compared with non-walkers, as suggested by Giles-Corti and Donovan [42] and Titze *et al. *[22] who also reported similarly unintuitive findings. Alternatively, it may represent a genuine association whereby walking to work is more prevalent in built up areas which have higher traffic levels. Further analyses in this study will draw on secondary data sources and GIS to explore the associations between travel behaviour and more objective assessments of the environment on and along the route to work.

In contrast to much of the literature which reports positive associations between psychological measures and walking or cycling behaviours [14,40], we found that few of the psychological measures related to car use were negatively associated with walking and cycling. Favourable attitudes towards car use were positively associated with walking. The reason for this finding is not apparent, although it is possible that these measures captured the potential enjoyment of using the car, rather than the practical feasibility of doing so. For example, it may be possible for a participant to respond positively to an item assessing attitude towards car use, but also to respond negatively to an item assessing perceived behavioural control if it is not practical for that person to use a car. Alternatively, it may be that participants who reported walking and reported positive attitudes towards car use did so because they travel to work using a combination of driving and walking. Again, the reasons are not immediately apparent but it may be that enjoyment of cycling and car use co-exist. This hypothesis appears feasible given the local context of the study, in which the locations of park-and-ride sites facilitate the use of combinations of travel modes and in which cycling is socially patterned in such a way that relatively affluent individuals can afford to own a car but also to live close enough to work to cycle. A mixed method exploration of how and why people commute by car in this sample will be the subject of a further paper.

Previous research has also highlighted the distance to work as an important explanatory variable for both walking and cycling behaviours. Interestingly, in this study the effects of distance on walking in particular were much stronger in the subset without access to a car. It may be that those participants who have no access to a car walk all the way to work, whereas those who do have access to a car may walk as part of a longer journey, for example by using off-site car parks or park-and-ride sites which encourage walking for short distances. This may explain why distance between home and work is more strongly associated with travel behaviour in the subgroup without access to a car than in those with access to a car. However, given the relatively small percentage of participants who did not have access to a car, the analysis may have been underpowered to detect an association in this group, regardless of the explanatory variable under consideration. This opportunity to combine travel modes is relatively common in older cities in the UK, such as Cambridge, Oxford and York, where the geography and historical development of the area means that the availability of parking in city centres is often limited. Analyses exploring the characteristics of those who walk or cycle in combination with other travel modes as part of a longer journey will be subject of a further paper.

In summary, we found that individual and household characteristics, as well as perceptions of the route environment and psychological measures related to car use, were associated with walking and cycling to and from work; however the contribution of these explanatory variables to behaviour was relatively small and the individual and household characteristics explained more of the total variance in cycling than the other variables. This raises two important points. First, it identifies a need for greater consideration of the range of factors which influence behaviour and the interactions between them. It is likely that behavioural choices, particularly about travel to and from work, are made in the context of wider consideration of the needs and requirements of other people [43], especially other members of the household but also those from work or other social settings. Researchers should therefore consider moving beyond asking individuals to reflect on their own views and perceptions towards attempts to ascertain the social and physical contexts of travel decision-making, for example by using a combination of quantitative and qualitative methods to gain a greater understanding of the role of household, neighbourhood and workplace social contexts in shaping behaviour. Second, although perceptions of the route and psychological measures related to car use made a relatively modest contribution to the models, these factors do appear to have a role in explaining patterns of walking and cycling to work, especially in those with access to a car. Understanding the reasons for these associations in particular subgroups and how changes in perceptions of the environment or psychological orientation towards car use could be brought about is important, but it is likely that both psychological and environmental influences on travel behaviour will need to be tackled in order to bring about sustained behaviour change in the population as a whole [44].

## Conflict of interests

The authors declare that they have no competing interests.

## Authors' contributions

JP and DO conceived of the study and wrote the manuscript. JP conducted the analysis and drafted the manuscript. SG, AJ and RM advised on the design of the study and the interpretation of the emerging findings and contributed to the critical revision of the paper. All authors read and approved the final manuscript.

## Supplementary Material

Additional file 1 **Survey Questionnaire**. Questionnaire used in study. Click here for file

Additional file 2 **Description and distribution of psychological measures towards regarding car use and route perceptions**. Description and distribution of psychological measures towards regarding car use and route perceptions. Click here for file
