# Supplementary material for: Correlates of time spent walking and cycling to and from work: baseline results from the commuting and health in Cambridge study
Source: Int J Behav Nutr Phys Act. 2011 Nov 10;8:124. doi: 10.1186/1479-5868-8-124 (PMC3254135; doi:10.1186/1479-5868-8-124)
Supplement: Additional file 1 — Survey Questionnaire. Questionnaire used in study. [file 1479-5868-8-124-S1.PDF]

MRC | Epidemiology Unit

Commuting and health in Cambridge Questionnaire 2009

About this questionnaire

This questionnaire booklet has two parts.

Part 1 is a **Recent Physical Activity Questionnaire**. This is designed to find out about your physical activity in your everyday life during the last four weeks. It is divided into three sections:

- Section A asks about your physical activity patterns in and around the house
- Section B asks about your travel to work and your activity at work
- Section C asks about your recreational activities.

Part 2 is a **travel and general questionnaire**. This is designed to find out about your general health, your travel patterns, your views, and some background information about yourself.

YOUR ANSWERS WILL BE TREATED AS STRICTLY CONFIDENTIAL

How to complete the questionnaire

The questionnaire should take about 20 minutes to complete. Please try to answer every question. Please use a blue or black pen.

Some questions ask you to **tick** a box. Please tick the box that applies to you.

Example: Are you male or female? Male ☑ Female ☐

Other questions ask you to **write numbers** in a box.

Example: What is your age? *Write in* [53] years

Don't worry if you make a **mistake** — just cross out the mistake and put in the correct answer.

Example: Do you have access to a bicycle? Yes ☒ No ☑

PART 1: RECENT PHYSICAL ACTIVITY QUESTIONNAIRE

Section A — Home activities

Getting about

1 Which form of transport have you used most often in the last four weeks apart from your journey to and from work?

Tick one only

- Car / motor vehicle ☐
- Walking ☐
- Public transport ☐
- Cycling ☐

TV, DVD or video viewing

Average over the last four weeks

2 Hours of TV, DVD or video watched per day

Tick one box on each line

	None	Less than 1 hour a day	1 to 2 hours a day	2 to 3 hours a day	3 to 4 hours a day	More than 4 hours a day
On a weekday before 6 pm	☐	☐	☐	☐	☐	☐
On a weekday after 6 pm	☐	☐	☐	☐	☐	☐
On a weekend day before 6 pm	☐	☐	☐	☐	☐	☐
On a weekend day after 6 pm	☐	☐	☐	☐	☐	☐

Computer use at home but not at work (e.g. internet, email, Playstation, Xbox, Gameboy, etc.)

Average over the last four weeks

3 Hours of home computer use per day

Tick one box on each line

	None	Less than 1 hour a day	1 to 2 hours a day	2 to 3 hours a day	3 to 4 hours a day	More than 4 hours a day
On a weekday before 6 pm	☐	☐	☐	☐	☐	☐
On a weekday after 6 pm	☐	☐	☐	☐	☐	☐
On a weekend day before 6 pm	☐	☐	☐	☐	☐	☐
On a weekend day after 6 pm	☐	☐	☐	☐	☐	☐

Stair climbing at home

Average over the last four weeks

4 Number of times you climbed up a flight of stairs (approx. 10 steps) each day at home

Tick one box on each line

	None	1 to 5 times a day	6–10 times a day	11–15 times a day	16–20 times a day	More than 20 times a day
On a weekday	☐	☐	☐	☐	☐	☐
On a weekend day	☐	☐	☐	☐	☐	☐

Section B — Activity at work

Please answer this section to describe if you have been in paid employment at any time during the last four weeks or you have done regular, organised voluntary work.

5 Have you been in employment during the last four weeks?

Tick one only Yes ☐ No ☐

6 During the last four weeks how many hours work did you do per week?

Write in number
If none, write '0'

Four weeks ago ☐

Three weeks ago ☐

Two weeks ago ☐

Last week ☐

Type of work

7 We would like to know the type and amount of physical activity involved in your work. Please tick the option that best corresponds with your occupation(s) in the last four weeks from the following four possibilities:

Tick one only

1. **Sedentary occupation**
You spend most of your time sitting (such as in an office) ☐

2. **Standing occupation**
You spend most of your time standing or walking. However, your work does not require intense physical effort (e.g. shop assistant, hairdresser, guard) ☐

3. Manual work
This involves some physical effort including handling of heavy objects and use of tools (e.g. plumber, electrician, carpenter) ☐

4. Heavy manual work
This implies very vigorous physical activity including handling of very heavy objects (e.g. dock worker, miner, bricklayer, construction worker) ☐

Travel to and from work in the last four weeks

8 What is the approximate distance from your home to your work?

Write in ☐☐☐ miles **OR** ☐☐☐ km

9 How many times a week did you travel from home to your main work? Count outward journeys only.

Write in number
If none, write '0' ☐

10 How did you normally travel to work?

Tick one box on each line	Always	Usually	Occasionally	Never or rarely
By car or motor vehicle	☐	☐	☐	☐
By works or public transport	☐	☐	☐	☐
By bicycle	☐	☐	☐	☐
Walking	☐	☐	☐	☐

11 What is the postcode for your main place of work DURING THE LAST 4 WEEKS?

Write in [ ][ ][ ][ ][ ][ ][ ]

12 If not known
please give your work address

13 What is the postcode for your home address?

Write in [ ][ ][ ][ ][ ][ ][ ]

Section C — Recreation

The following questions ask about how you spent your leisure time.

Please indicate how often you did each activity on average over the last four weeks

Please indicate the average length of time that you spent doing the activity on each occasion.

Example: If you went walking for pleasure for 40 minutes once a week, and if you did weeding or pruning every fortnight and took 1 hour and 10 minutes on each occasion, you would complete the table below as follows:

Please give an answer for the NUMBER OF TIMES you did the following activities in the past four weeks and the AVERAGE TIME you spent on each activity

	**Number of times you did the activity in the last four weeks**							**Average time per episode**	
Please complete EACH line	None	Once in the last 4 weeks	2 to 3 times in the last 4 weeks	Once a week	2 to 3 times a week	4 to 5 times a week	Every day	Hours	Mins
Weeding and pruning			✓					1	10
Walking for pleasure				✓					40

Now complete the table on pages 6 and 7

14 Please give an answer for the NUMBER OF TIMES you did the following activities in the past four weeks and the AVERAGE TIME you spent on each activity

Please complete EACH line	None	Once in the last 4 weeks	2 to 3 times in the last 4 weeks	Once a week	2 to 3 times a week	4 to 5 times a week	Every day	Hours	Mins
	**Number of times you did the activity in the last four weeks**							**Average time per episode**	
Swimming — competitive	☐	☐	☐	☐	☐	☐	☐	☐	☐
Swimming — leisurely	☐	☐	☐	☐	☐	☐	☐	☐	☐
Backpacking or mountain climbing	☐	☐	☐	☐	☐	☐	☐	☐	☐
Walking for pleasure (not as a means of transport)	☐	☐	☐	☐	☐	☐	☐	☐	☐
Racing or rough terrain cycling	☐	☐	☐	☐	☐	☐	☐	☐	☐
Cycling for pleasure (not as a means of transport)	☐	☐	☐	☐	☐	☐	☐	☐	☐
Mowing the lawn	☐	☐	☐	☐	☐	☐	☐	☐	☐
Watering the lawn or garden	☐	☐	☐	☐	☐	☐	☐	☐	☐
Digging, shovelling or chopping wood	☐	☐	☐	☐	☐	☐	☐	☐	☐
Weeding or pruning	☐	☐	☐	☐	☐	☐	☐	☐	☐
DIY, e.g. carpentry, home or car maintenance	☐	☐	☐	☐	☐	☐	☐	☐	☐
High impact aerobics or step aerobics	☐	☐	☐	☐	☐	☐	☐	☐	☐
Other types of aerobics	☐	☐	☐	☐	☐	☐	☐	☐	☐
Exercise with weights	☐	☐	☐	☐	☐	☐	☐	☐	☐
Conditioning exercises, e.g. using a bike or rowing machine	☐	☐	☐	☐	☐	☐	☐	☐	☐
Floor exercises, e.g. stretching, bending, keep fit or yoga	☐	☐	☐	☐	☐	☐	☐	☐	☐
Dancing, e.g. ballroom or disco	☐	☐	☐	☐	☐	☐	☐	☐	☐

Please give an answer for the NUMBER OF TIMES you did the following activities in the past four weeks and the AVERAGE TIME you spent on each activity

	**Number of times you did the activity in the last four weeks**							**Average time per episode**	
Please complete EACH line	None	Once in the last 4 weeks	2 to 3 times in the last 4 weeks	Once a week	2 to 3 times a week	4 to 5 times a week	Every day	Hours	Mins
Competitive running	☐	☐	☐	☐	☐	☐	☐	☐	☐
Jogging	☐	☐	☐	☐	☐	☐	☐	☐	☐
Bowling — indoor, lawn or ten pin	☐	☐	☐	☐	☐	☐	☐	☐	☐
Tennis or badminton	☐	☐	☐	☐	☐	☐	☐	☐	☐
Squash	☐	☐	☐	☐	☐	☐	☐	☐	☐
Table tennis	☐	☐	☐	☐	☐	☐	☐	☐	☐
Golf	☐	☐	☐	☐	☐	☐	☐	☐	☐
Football, rugby or hockey	☐	☐	☐	☐	☐	☐	☐	☐	☐
Cricket	☐	☐	☐	☐	☐	☐	☐	☐	☐
Rowing	☐	☐	☐	☐	☐	☐	☐	☐	☐
Netball, volleyball or basketball	☐	☐	☐	☐	☐	☐	☐	☐	☐
Fishing	☐	☐	☐	☐	☐	☐	☐	☐	☐
Horse-riding	☐	☐	☐	☐	☐	☐	☐	☐	☐
Snooker, billiards or darts	☐	☐	☐	☐	☐	☐	☐	☐	☐
Musical instrument playing or singing	☐	☐	☐	☐	☐	☐	☐	☐	☐
Ice skating	☐	☐	☐	☐	☐	☐	☐	☐	☐
Sailing, wind-surfing or boating	☐	☐	☐	☐	☐	☐	☐	☐	☐
Martial arts, boxing or wrestling	☐	☐	☐	☐	☐	☐	☐	☐	☐

PART 2: TRAVEL AND GENERAL QUESTIONNAIRE

About your health

15 Do you have any long-term illness, health problem or disability which limits your daily activities or the work you can do? Include problems which are due to old age.

Tick one only Yes ☐ No ☐

16 Do you have any difficulty walking for a quarter of a mile on the level?

Tick one only Yes ☐ No ☐

17 In the PAST TWELVE MONTHS how many days were you off sick for health reasons?

Write in number
If none, write '0' ☐

18 How tall are you? (with your shoes off)

Write in ☐ ft ☐ in **OR** ☐ cm

19 How much do you weigh? (in light indoor clothes)

Write in ☐ st ☐ lb **OR** ☐ kg

The next section asks for your views about your health. This information will help keep track of how you feel and how well you are able to do your usual activities. For each of the following questions, please tick the one box that best describes your answer.

20 Overall, how would you rate your health during the PAST FOUR WEEKS?

Excellent	Very good	Good	Fair	Poor	Very poor
☐	☐	☐	☐	☐	☐

21 During the PAST FOUR WEEKS, how much did physical health problems limit your usual physical activities (such as walking or climbing stairs)?

Not at all	Very little	Somewhat	Quite a lot	Could not do physical activities
☐	☐	☐	☐	☐

22 During the PAST FOUR WEEKS, how much difficulty did you have doing your daily work, both at home and away from home, because of your physical health?

None at all	A little bit	Some	Quite a lot	Could not do daily work
☐	☐	☐	☐	☐

23 How much BODILY pain have you had during the PAST FOUR WEEKS?

None	Very mild	Mild	Moderate	Severe	Very severe
☐	☐	☐	☐	☐	☐

24 During the PAST FOUR WEEKS, how much energy did you have?

Very much	Quite a lot	Some	A little	None
☐	☐	☐	☐	☐

25 During the PAST FOUR WEEKS, how much did your physical health or emotional problems limit your usual social activities with family or friends?

Not at all	Very little	Somewhat	Quite a lot	Could not do social activities
☐	☐	☐	☐	☐

26 During the PAST FOUR WEEKS, how much have you been bothered by emotional problems (such as feeling anxious, depressed or irritable)?

Not at all	Slightly	Moderately	Quite a lot	Extremely
☐	☐	☐	☐	☐

27 During the PAST FOUR WEEKS, how much did personal or emotional problems keep you from doing your usual work, school or other daily activities?

Not at all	Very little	Somewhat	Quite a lot	Could not do daily activities
☐	☐	☐	☐	☐

SF-8™ 4-Week Recall Version — © 1999-2001 — QualityMetric, Inc. — All rights reserved

About your travel options

28 How many cars or vans are owned, or available for use, by members of your household?
Do not include motorcycles, scooters or mopeds.

Write in number
If none, write '0' ☐

29 Do you hold a full driving licence valid in Great Britain either to drive a car or to drive a motorcycle, scooter or moped?

Tick one only Yes ☐ No ☐

30 Do you have access to car parking at your place of work? This includes parking anywhere on the site, for example in a multi-storey car park. It does not include parking on the streets nearby or at a park-and-ride.

Tick one only

Yes, and I have to pay to park there ☐

Yes, and I do not have to pay to park there ☐

No ☐

31 Do you ever travel by car for part or all of the journey to or from work?
This includes as a passenger in a car driven by someone else.

Tick one only Yes ☐ → ***Go to Q. 32*** No ☐ → ***Go to Q. 34***

Thinking about the car you are most likely to use to travel to and from work:

32 What type of fuel does the car use?

Tick one only

Petrol ☐

Diesel ☐

Hybrid or other ☐

33 What is the engine size of the car?

Write in ☐ cc **OR** ☐ litres

34 Do you have access to a bicycle?

Tick one only Yes ☐ No ☐

35 Do you ever cycle part or all of the journey to or from work?
This includes cycling to or from a bus stop, railway station or park-and-ride.

Tick one only Yes ☐ → ***Go to Q. 36*** No ☐ → ***Go to Q. 37***

36 How long does the cycling part of the journey usually take? ☐ minutes each way

37 Do you ever walk part or all of the journey to or from work?
This includes walking to or from a bus stop, railway station or park-and-ride.

Tick one only Yes ☐ → ***Go to Q. 38*** No ☐ → ***Go to Q. 39***

38 How long does the walking part of the journey usually take? ☐ minutes each way

About your travel to and from work in the last seven days

In this section, we are interested in **how you travelled to and from work on each of the last seven days**.

39 **For each of the last seven days, please tell us what time you started and finished work and tick all the modes of transport you used on the journey to and from work.** If you did not travel to work on a particular day (either because it was a day off or because you worked at home), please tick the box 'Did not travel to work'. If your journey to and from work was the same on more than one day, you can tick the box 'Same as previous' instead of repeating the information again. *We have given you an example for one day in the first row of the table.*

Which modes of transport did you use on this journey? *Tick all that apply*

Day of the week	Time started work	Time finished work	Did not travel to work		Same as previous	Bus or coach	Train or underground	Car, taxi or van	Motorcycle or moped	Bicycle	Walking	Other
Thu	7.30 am	3.30 pm	☐	**To work**		☑	☐	☐	☐	☐	☑	☐
				**From work**		☑	☐	☐	☐	☐	☑	☐
			☐	**To work**		☐	☐	☐	☐	☐	☐	☐
				**From work**		☐	☐	☐	☐	☐	☐	☐
			☐	**To work**	☐	☐	☐	☐	☐	☐	☐	☐
				**From work**	☐	☐	☐	☐	☐	☐	☐	☐
			☐	**To work**	☐	☐	☐	☐	☐	☐	☐	☐
				**From work**	☐	☐	☐	☐	☐	☐	☐	☐
			☐	**To work**	☐	☐	☐	☐	☐	☐	☐	☐
				**From work**	☐	☐	☐	☐	☐	☐	☐	☐
			☐	**To work**	☐	☐	☐	☐	☐	☐	☐	☐
				**From work**	☐	☐	☐	☐	☐	☐	☐	☐
			☐	**To work**	☐	☐	☐	☐	☐	☐	☐	☐
				**From work**	☐	☐	☐	☐	☐	☐	☐	☐
			☐	**To work**	☐	☐	☐	☐	☐	☐	☐	☐
				**From work**	☐	☐	☐	☐	☐	☐	☐	☐

About all the journeys you made yesterday

In this section, we are interested in more detail about **all the journeys you made yesterday** (between 3 a.m. yesterday and 3 a.m. today).

40 **Please list each journey you made yesterday to get from place to place.** These might include, for example, going to work, going out to get lunch, coming home from work, going shopping, going to the doctor's, visiting friends, or escorting someone else (e.g. taking a child to school). Please include time spent travelling on foot or by bike, even if this was part of a longer journey (e.g. by bus or train). But please **do not include** journeys you made as part of your job (e.g. as a delivery driver), or walking or cycling purely for recreation or exercise (e.g. walking the dog).

*We have given you an example of **one** journey. This person walked for ten minutes to the bus stop, rode on the bus for 22 minutes, and then walked for five minutes to get to work (a total of 15 minutes walking). They did not count the time spent waiting for the bus.*

	Office use only	**What was the purpose of the journey?** Please give a simple description, e.g. 'to work', 'to get home from work', 'shopping', 'take child to school'	**How many MINUTES did you spend TRAVELLING by each mode of transport on this journey?** Do not count time spent waiting for buses, trains etc. Bus or coach	Train or underground	Car, taxi or van	Motorcycle or moped	Bicycle	Walking	Other
		To work	22					15	
**Journey 1**									
**Journey 2**									
**Journey 3**									
**Journey 4**									

Continue over the page if necessary

About all the journeys you made yesterday (continued)

		**What was the purpose of the journey?** Please give a simple description, e.g. 'to work', 'to get home from work', 'shopping', 'take child to school'	**How many MINUTES did you spend TRAVELLING by each mode of transport on this journey?** Do not count time spent waiting for buses, trains etc.						
			Bus or coach	Train or underground	Car, taxi or van	Motorcycle or moped	Bicycle	Walking	Other
**Journey 5**									
**Journey 6**									
**Journey 7**									
**Journey 8**									
**Journey 9**									
**Journey 10**									
**Journey 11**									
**Journey 12**									

About your views on travelling to and from work

For each of the following statements about your journey to and from work, please tick one box to show how strongly you agree or disagree.

Tick one per row

**41 On my journey to and from work:**	Strongly agree	Agree	Neither agree nor disagree	Disagree	Strongly disagree
It is pleasant to walk	☐	☐	☐	☐	☐
The roads are dangerous for cyclists	☐	☐	☐	☐	☐
There is convenient public transport	☐	☐	☐	☐	☐
There are convenient routes for cycling	☐	☐	☐	☐	☐
There is little traffic	☐	☐	☐	☐	☐
There are no convenient routes for walking	☐	☐	☐	☐	☐
It is safe to cross the road	☐	☐	☐	☐	☐
**42 For me, to get to and from work next time:**					
Overall, it would be good to use a car	☐	☐	☐	☐	☐
Most people who are important to me would support my using a car	☐	☐	☐	☐	☐
It would be easy for me to use a car	☐	☐	☐	☐	☐
I intend to use a car	☐	☐	☐	☐	☐
It would be pleasant to use a car	☐	☐	☐	☐	☐
Most people who are important to me think I should use a car	☐	☐	☐	☐	☐
I would be able to use a car	☐	☐	☐	☐	☐
I am likely to use a car	☐	☐	☐	☐	☐

For each of the following statements about your journey to and from work, please tick one box to show how strongly you agree or disagree.

Tick one per row

**43 Using a car to get to and from work is something:**	Strongly agree	Agree	Neither agree nor disagree	Disagree	Strongly disagree
I do frequently	☐	☐	☐	☐	☐
I do automatically	☐	☐	☐	☐	☐
that would require effort not to do	☐	☐	☐	☐	☐
that belongs to my daily routine	☐	☐	☐	☐	☐
I would find hard not to do	☐	☐	☐	☐	☐
that's typically 'me'	☐	☐	☐	☐	☐
I have been doing for a long time	☐	☐	☐	☐	☐

About you and your household

44 Are you male or female? *Tick one only* Male ☐ Female ☐

45 What is your date of birth? *Write in* ☐ / ☐ / ☐

date *month* *year*

46 What is your highest educational qualification?

Tick one only

Degree, NVQ4, NVQ5 or equivalent	☐
BTEC (Higher), BEC (Higher), TEC (Higher), HNC, HND or equivalent	☐
GCE 'A' Level, NVQ3, Scottish Higher or equivalent	☐
BTEC (National), TEC (National), BEC (National), ONC, OND or equivalent	☐
GCSE Grades A to C, GCSE 'O' Level, CSE Grade 1, NVQ2 or equivalent	☐
Other qualifications	☐
No formal qualifications	☐

47 How many other people live in your household?
We mean people who have your accommodation as their only or main residence, and who either share at least one meal a day with you or share the living accommodation (living room or sitting room) with you.

Write in number
If none, write '0'

Children aged under 5 ☐

Children aged between 5 and 15 ☐

Adults aged 16 and over (do not include yourself) ☐

48 Does your household own or rent its accommodation?

Tick one only

Rents it from the council, a housing association, or a charity ☐

Rents it from a private landlord or letting agency ☐

Partly owns it and partly rents it (shared ownership) ☐

Owns it (including buying with a mortgage) ☐

Other ☐

Finally

49 Please enter today's date. *Write in* ☐ ☐ / ☐ / 09

day of the week *date* *month*

THANK YOU VERY MUCH FOR TAKING PART IN THIS STUDY
