# Supplementary material for: Correlates of time spent walking and cycling to and from work: baseline results from the commuting and health in Cambridge study
Source: Int J Behav Nutr Phys Act. 2011 Nov 10;8:124. doi: 10.1186/1479-5868-8-124 (PMC3254135; doi:10.1186/1479-5868-8-124)
Supplement: Additional file 2 — Description and distribution of psychological measures towards regarding car use and route perceptions. Description and distribution of psychological measures towards regarding car use and route perceptions. [file 1479-5868-8-124-S2.PDF]

Additional file 2: Description and distribution of psychological measures regarding car use and route perceptions

Variables and items	Categorisation	Percentage (n)
**Psychological measures towards car use** [1]		
Mean intention score (range of scores 1-5)		
I am likely to use a car	Low intention<br>Mid intention<br>High intention	Approximating to tertiles
I intend to use a car		
Mean attitude score (range of scores 1-5)		
It would be pleasant to use a car	Low attitude<br>Mid attitude<br>High attitude	
Overall, it would be good to use a car		
Mean PBC score (range of scores 1-5)		
I would be able to use a car	Low PBC<br>Mid PBC<br>High PBC	
It would be easy for me to use a car		
Mean social norm score (range of scores 1-5)		
Most people who are important to me would support my using a car	Low social norm<br>Mid social norm<br>High social norm	
Most people who are important to me think I should use a car		
**Habit measure** (Mean of scores 1-5)<br>Using a car to get to and from work is something…		
I do automatically		
that would require effort not to do		
that belongs to my daily routine	Low habit ($\leq$1)<br>High habit (>1)	40.3 (461)<br>59.7 (684)
I would find hard not to do		
that's typically 'me'		
**Perceptions of the route to work** [2, 3]		
It is pleasant to walk	Disagree<br>Agree	43.9 (497)<br>56.1 (636)
There is convenient public transport	Disagree<br>Agree	61.5 (706)<br>38.5 (441)
There is little traffic	Disagree<br>Agree	89.7 (1035)<br>10.3 (119)
There are no convenient routes for walking	Disagree<br>Agree	75.9 (871)<br>24.1 (276)
It is safe to cross the road	Disagree<br>Agree	50.3 (579)<br>49.7 (571)
The roads are dangerous for cyclists	Disagree<br>Agree	42.1 (484)<br>57.9 (666)
There are convenient routes for cycling	Disagree<br>Agree	44.0 (508)<br>56.0 (645)

All items were answered on a 5 point scale.
Stems of questions were as follows: [1] "For me, to get to and from work next time: " [2] "On my journey to and from work:" [3]Responses to these items were collapsed such that those who "strongly agreed" or "agreed" were compared with those who "strongly disagreed", "disagreed" or "neither disagreed or agreed".
